# Comparison of background EEG activity of different groups of patients with idiopathic epilepsy using Shannon spectral entropy and cluster-based permutation statistical testing

**DOI:** 10.1371/journal.pone.0184044

**Published:** 2017-09-18

**Authors:** Jose Antonio Urigüen, Begoña García-Zapirain, Julio Artieda, Jorge Iriarte, Miguel Valencia

**Affiliations:** 1 Deustotech-Life (eVida), Universidad de Deusto, Bilbao, Spain; 2 Clínica Universidad de Navarra (CUN), Universidad de Navarra, Pamplona, Spain; 3 Centro de Investigación Médica Aplicada (CIMA), Universidad de Navarra, Pamplona, Spain; 4 Instituto de Investigación Sanitaria de Navarra (IdiSNA), Pamplona, Spain; Radboud Universiteit, NETHERLANDS

## Abstract

Idiopathic epilepsy is characterized by generalized seizures with no apparent cause. One of its main problems is the lack of biomarkers to monitor the evolution of patients. The only tools they can use are limited to inspecting the amount of seizures during previous periods of time and assessing the existence of interictal discharges. As a result, there is a need for improving the tools to assist the diagnosis and follow up of these patients. The goal of the present study is to compare and find a way to differentiate between two groups of patients suffering from idiopathic epilepsy, one group that could be followed-up by means of specific electroencephalographic (EEG) signatures (intercritical activity present), and another one that could not due to the absence of these markers. To do that, we analyzed the background EEG activity of each in the absence of seizures and epileptic intercritical activity. We used the Shannon spectral entropy (SSE) as a metric to discriminate between the two groups and performed permutation-based statistical tests to detect the set of frequencies that show significant differences. By constraining the spectral entropy estimation to the [6.25–12.89) Hz range, we detect statistical differences (at below 0.05 alpha-level) between both types of epileptic patients at all available recording channels. Interestingly, entropy values follow a trend that is inversely related to the elapsed time from the last seizure. Indeed, this trend shows asymptotical convergence to the SSE values measured in a group of healthy subjects, which present SSE values lower than any of the two groups of patients. All these results suggest that the SSE, measured in a specific range of frequencies, could serve to follow up the evolution of patients suffering from idiopathic epilepsy. Future studies remain to be conducted in order to assess the predictive value of this approach for the anticipation of seizures.

## Introduction

Epilepsy is a disorder of the brain characterized by an enduring predisposition to generate unpredictable seizures. It is one of the most common neurological disorders affecting approximately 1 in 200 people. About 70% of patients are usually well controlled with antiepileptic drugs, but still 30% of them show seizures that are resistant to medication and continue suffering from them [1].

Within the spectra of epileptic syndromes, primary generalized epilepsy or idiopathic epilepsy is characterized by generalized seizures with no apparent cause. The general idea is that they are genetic and not caused by any brain physical abnormality as the brain is anatomically normal. One of the main problems in the management of these patients is the lack of reliable biomarkers or the impossibility to predict the occurrence of new seizures. Diagnosis is based on parameters like frequency of occurrence, severity and characteristics of the seizures. Tools such as the electroencephalogram (EEG) [2] offer excellent temporal and spatial resolutions for the assessment of brain activity and their study allows to characterize the seizures, frequently detects the presence of abnormal inter-ictal activities (such as spikes, spike-wave complexes, sharp waves, or mono-rhythmic activity [3]) and provides important parameters that are used as prognostic information to guide the therapeutic treatment. Nevertheless, seizures and epileptiform transients are not always present in EEG recordings and, in such situations, the development of techniques that help evaluate and follow up epileptic patients may be critical. To date, differentiating subjects by visual inspection or by applying signal processing methods on background EEG activities is still quite unreliable, mainly within the clinical framework. Thus, no consistent markers exist and therefore a more precise prevention and follow up is not possible.

EEG analysis has been approached from different perspectives; traditionally, basic linear analyses in time and frequency domains were used. More recently, and motivated by the studies about the inherent non-linearity of the brain, some advanced features have been proposed [4, 5]. Changes in EEG signal such as peak-to-peak amplitude, distance and energy ratio between seizure and non-seizure intervals or entropy of ictal states versus baseline periods have been used as metrics for the evaluation of epileptic activity [6, 7]. However, parameters are often developed or selected under customized and case-specific schemes and thus lack the suitability to be generalized. Here we focus on Shannon spectral entropy (SSE), an entropy-based measure widely used as a quantifier of the complexity of a signal [8]. As it does not depend on absolute scales like the amplitude or the frequency of the signal, it is well suited to deal with the variability of EEG rhythms across and among subjects.

In this study, we analyze background EEG activity of patients suffering from primary generalized epilepsy. Segments of EEGs selected for the analyses did not include interictal activities and were indistinguishable from normal recordings. Nevertheless, the first set of data was obtained from video-EEG recordings of patients that contained epileptiform activities (epileptic markers) in other portions of their files. In contrast, the second set of data was obtained from video-EEG recordings of patients that presented no epileptic markers for the whole length of their files, thus hindering clinical evaluation. We employed the SSE together with a cluster-based statistical framework to identify the set of frequencies that differentiate such sets of data and find that the SSE computed in the [6.25–12.89)Hz range allows to separate (or classify) these two group of patients. Interestingly, we observe that, measured in such range, this local spectral entropy (LSE) estimate shows a trend that is inversely related to the elapsed time from the last seizure and, more importantly, that it approaches asymptotically to the values shown by a control group of healthy subjects monitored in the same conditions.

## Materials and methods

### Subjects

We study EEG recordings from 20 patients with generalized epilepsy who had been monitored in our video-EEG Unit at the Clínica Universidad de Navarra. The recordings correspond to interictal periods. Patients comprise 14 women (f) and 6 men (m), aged between 11 and 70. Among them, 16 have primary generalized epilepsy (pge), 1 suffers from primary generalized epilepsy with hyperactivity (pge+h) and 3 where diagnosed of likely suffering from primary generalized epilepsy (lpge). Clinical, EEG and treatment features of epileptic patients are summarized in Table 1. In addition, a control group of 10 healthy subjects with no history of neurological or psychiatric disorders were also investigated (3 women and 7 men, aged between 23 and 60).

**Table 1:** Group 1 include the patients with abnormal EEG (although we analyze only normal periods).

File	Sex	Group	Age	Epilepsy	Drugs	Days from last crisis
a1	f	1	23	pge	keppra, gardenal	30
b3	f	1	22	pge	keppra, gardenal	13
b5	f	1	31	pge	topamax, lamictal, rivotril	20
b7	m	1	16	pge	depakine, concerta	365
c3	f	1	15	lpge	depakine, lamictal	6
c5	f	1	60	pge	luminal, noiafren	1
c7	f	1	21	pge	zonegran, kepra	1
d7	f	1	47	pge	depakine, etoxusimida, rivotril, escitalopram	30
g3	m	1	67	pge	topiramato, rivotril	300
g5	f	1	22	pge	no	1
c2	f	2	32	lpge	keppra, zebinix	
d6	f	2	70	pge	fenobarbital	2555
e8	f	2	51	lpge	zonegran, carbamazepina	45
e12	m	2	23	pge	keppra	30
1-epgp-normal	f	2	19	pge	clonzacepam, zonegram, lamotrigina	500
2-epgp-normal	f	2	20	pge	clonzacepam, zonegram, lamotrigina	1800
3-epgp-normal	f	2	16	egp	lamotrigina	3285
4-epgp-normal	m	2	36	pge	topiramato	1825
5-epgp-normal	m	2	11	pge	no treatment	1825
6-epgp-normal	m	2	15	pge	valproato	730

Group 2 include patients with fully normal EEG. Here, pge stands for primary generalized epilepsy and lpge for likely suffering from primary generalized epilepsy.

The study was carried out in a retrospective way, by using data coming from routine tests conducted in the Hospital following clinical instruction. Every patient signed an informed consent indicating that recorded signals could be stored and later on used for teaching or research purposes after being properly anonymized. As a result, the use of this dataset does not need approval by an ethics committee but precludes their public sharing since no clause for data sharing was included (data could be available upon request to one of the specialist, J.I.). Only one of the authors (J.I.) had access to the personal data of the patients, but only in order to remove them from the recordings and to assign a random alphanumerical code to each final EEG file. Once patients were anonymized, they could not be identified by any means by none of the authors of the study, since they all merely had access to the final EEG files.

### EEG recordings

The EEGs were acquired by using standard video-EEG equipment [32-channel digital EEG with LaMont amplifiers (LaMont Medical, Madison, WI, U.S.A.) and Harmonie software (Stellate, Quebec Canada)]. The sampling frequency was 200 Hz and acquisition filters were set between 0.5 and 70 Hz. Electrodes were placed according to the 10–20 system and both mastoids were linked and used as reference. The duration of the segments is at least 120 s taken from interictal periods with no epileptiform patterns such as sharp waves, spikes, spike-wave complexes (spike-and-slow-wave complexes), nor polyspike-wave complexes. Activity was considered as normal and as such not representative of any neurological disease according to the evaluation of the two experts in the study (J.I and J.A.). Segments for the analyses were either obtained from recordings showing epileptiform activity in other portions of the video-EEG (epileptic patients group 1) or from recordings with no epileptiform signatures at all (epileptic patients group 2). Recordings for healthy subjects were acquired in the same conditions than that of the epileptic patients.

### Signal conditioning and preprocessing

**Fig 1:**
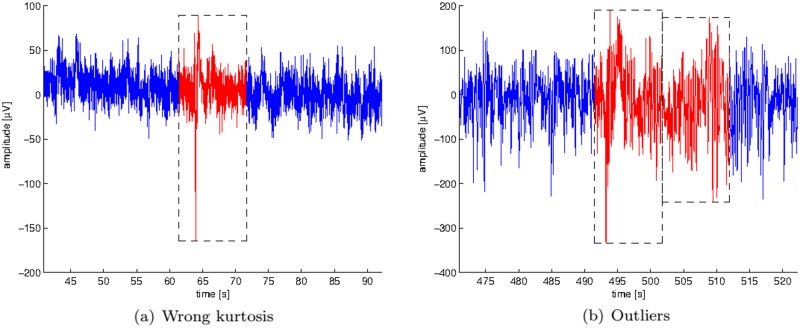
Epochs automatically marked to be rejected. The picture shows (a) one epoch (in red) of the first channel of one recorded EEG signal that does not satisfy the limits for kurtosis (rejkurt) used to identify background EEG signal; and (b) two epochs of the second channel of one recorded EEG signal that probably (jointprob) do not belong to background EEG signal.

EEG signals were bandpass filtered in between 0.5 and 70 Hz to cancel the main effect of physiological artifacts and epochs with evident signs of additional contamination were removed by means of an automatic approach for artifact rejection [2]. To do so, we first segmented the EEG signals into non-overlapping epochs of 10.24 seconds (2048 samples). Then epochs containing muscular, ocular or low-frequency artifacts were automatically detected by using the jointprob and rejkurt Matlab functions implemented in the EEGlab toolbox [9, 10]. Segments were marked as non-valid when they either appear as transient and unusual (i.e. their values were outliers relative to background activity), or when the absolute value of their kurtosis is too large compared to that of background activity [10]. We used thresholds equal to 3 times the standard deviations, which tend to work well in practice [10]. Fig 1 shows two examples of epochs automatically detected as artifacts and thus rejected for further analyses.

### Shannon spectral entropy and local spectral entropy

The Shannon spectral entropy can be used as an irregularity metric in terms of the *flatness of the spectrum*: if SSE is high it means that the spectrum tends to be broader and flatter, such as that of white noise; if it is low then the signal energy tends to be concentrated into few frequency bins, such as less complex signals or into specific frequencies such as sinusoids. [11]. In this way, the spectral entropy can quantify certain spectral patterns that correspond to intuitive visual differences in between regular and irregular signals.

The SSE for each individual epoch is obtained from the normalized power spectrum, defined by $S_{n}\left(f\right)=\frac{S(f)}{\sum S(f)}$ such that ∑*S*_*n*_(*f*) = 1, as follows:

$$\begin{array}{c} \text{SSE}=-\sum S_{n}\left(f\right)\log S_{n}\left(f\right), \end{array}$$ (1)

where all the sums comprise *only* the discrete bins of the frequency range for which the power spectrum exists (or is calculated). The base of the logarithm is 2 and in such case the units of SSE are bits. Note that, according to their definition *S*(*f*) and *S*_*n*_(*f*) are always greater than or equal to zero, the latter case contributing to zero in the sum Eq (1).

Even if the SSE has been widely used in similar contexts to ours, its calculation is usually conducted for the entire signal spectrum or restricted to predefined frequency bands [6, 7]. However, nothing stops us from defining arbitrary spectral intervals in which the spectral entropy might be of greater interest. Here we exploited such idea by defining the local spectral entropy as a metric to quantify the variation of the SSE with respect to the frequency. In order to detect frequencies that contribute to statistical significance in the comparison between the two epileptic groups, we splited the spectrum of interest into contiguous, possibly overlapping frequency bands and calculated the SSE at each window. We term this set of values the local spectral entropy (LSE) of the EEG signal.

**Fig 2:**
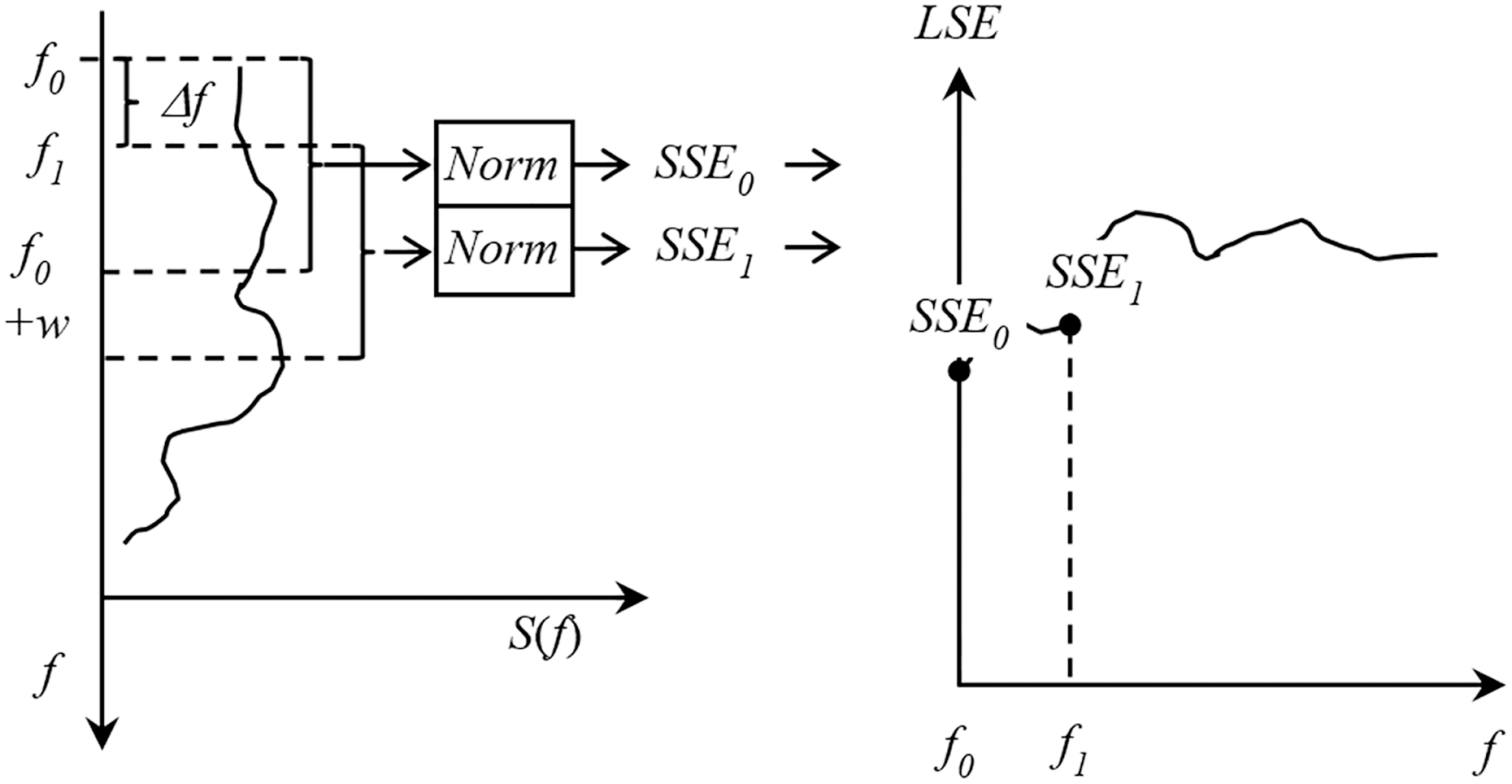
Local spectral entropy. Illustration of the proposed calculation of the LSE from the spectral entropy of consecutive frequency bins.

We define the LSE at frequency *f*_0_ as the SSE in the spectral window [*f*_0_, *f*_0_ + *w*), where *w* determines the window size and is user dependent. Then, given a power spectrum *S*(*f*) for frequencies [*f*_*a*_, *f*_*b*_), the LSE requires the calculation of the SSE in consecutive (or overlapping) windows of size *w* over the entire range of frequencies of interest [*f*_*a*_, *f*_*b*_), incrementing by Δ*f* at each step (note that if Δ*f* < *w*, then windows overlap). A practical example is shown in Fig 2.

The LSE allows us to consider those channels and frequencies at which the statistical effect might be observed are unknown in advance, which is something we cannot do with the SSE. Consequently, by comparing the LSE of the two groups of epileptic patients, our goal was to find statistical differences at specific frequency bands for one or more electrodes simultaneously.

In the experiments we estimated the power spectrum for every 10 second epoch of each subject (10 per group) and channel (19 per subject) via the spectopo Matlab function, implemented by the EEGlab toolbox. This function delivers an estimate of the power spectral density of the input by employing Welch’s overlapped segment averaging estimator. We used spectopo with a window size of 512 samples (which corresponds to an FFT length of 512) and 25% overlap. Then, we computed the set of values that comprise the LSE of each epoch from their power spectra and averaged them across epochs to characterize every subject and channel by a set of average LSE values. We used a window size *w* of 5Hz and a frequency step Δ*f* of 1 bin (1 bin equals 0.39Hz for an FFT of 512 points, at 200Hz) and calculated the LSE in between 0 and 45Hz. Thus, we obtained 115 values for the FFT of size 512, at the sampling frequency of 200Hz.

### Statistical analysis

Initially, an exploratory analysis was carried out to test the statistical properties of the data. Statistical tests revealed that the LSE values did not meet the normality (Kolmogorov—Smirnov test) and equality of variance (*F*-test) assumptions for parametric testing. Furthermore, and in order to control type I error when the multiplicity of testing is large (in our case 19 channels × 115 frequency values), a multiple comparison permutation approach was adopted [12, 13]. Importantly, this framework was also meant to detect the channels and frequency ranges at which the LSE may differ significantly among groups; values that are not known prior to applying the permutation test.

The single threshold test computes the permutation distribution of the maximal channel-frequency statistic [12] over the set of all channels and frequencies. This is based on the statistic obtained by comparing the LSE at each channel-frequency pair of the original configuration of subjects to the LSE at each channel-frequency pair of permuted configurations of subjects. However, rather than computing the single threshold distribution, we calculated the cluster threshold [12] distribution which is normally better suited to EEG and MEG data [13–15]. Specifically, we followed the approach given in [13], based on grouping together channel and frequency neighbours characterized by an individual test statistic smaller than a predefined threshold to form clusters. Then, a permutation test was conducted on the two groups by first performing random partitions of the original configuration of subjects, then obtaining clusters for the random partitions and finally comparing the cluster-level statistics with those of the original significant cluster. The proportion of random partitions that have a larger cluster-statistic than the observed one is called the permutation *p*-value. If it is smaller than the critical alpha-level, then it is possible conclude that the data in the two experimental conditions are significantly different.

### Data validation

Once the significant channel-frequency pairs were found, we evaluated the classification performance of the LSE for such frequency range by means of a two-class ROC (receiver operating characteristic) analysis. Classification statistics are summarized in terms of true sensitivity (true positive rate), specificity (true negative rate) and accuracy (total proportion of correct classification).

In addition to the ROC curves we performed a 5-fold cross-validation to determine the reproducibility of our experiments, using a 80/20 split. The basic idea consists in sequentially splitting the recordings so that each data point ends up in the 20% test set exactly once. Consequently, we divide the recordings of the two groups into sets of lengths 80%–20%, define the optimal ROC working point using 80% of the data and classify the other 20% based on the threshold associated to the optimal point.

To finish, and in order to assess the usefulness of the LSE, we compared the LSE values related to the recordings from both epileptic groups with those of the control group (healthy subjects). To do that, differences in the the averaged value of the LSE for the three different groups were assessed by using the Kruskal-Wallis non-parametric ANOVA followed by post-hoc multiple comparison tests.

## Results

### Statistical evaluation of the LSE metric

**Fig 3:**
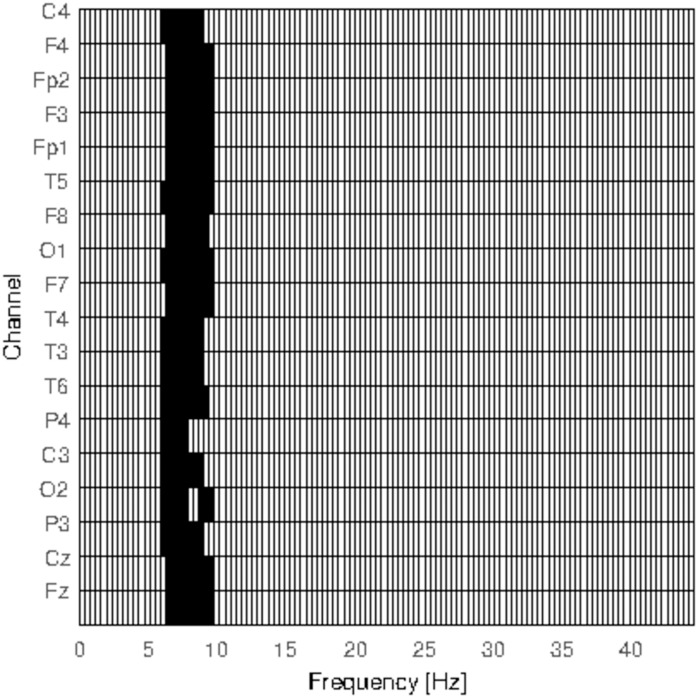
Clusters for the permutation test. Channel and frequency neighbors found by running the clustering stage of the permutation test for the local spectral entropy.

The first step of the permutation test consist of determining the channel-frequency clusters of the original configuration of subjects so that clusters corresponding to random partitions can be compared to the original ones and their significance be assessed. We used a sum of *t*-values as the cluster-level statistic. Fig 3 shows the clusters found prior to calculating the significance for the two groups of epileptic patients. The *x*-axis indicates the corresponding frequency and the *y*-axis the channel. There is only 1 main cluster, clearly visible in the figure, to which the clusters found when performing the permutations are compared.

**Fig 4:**
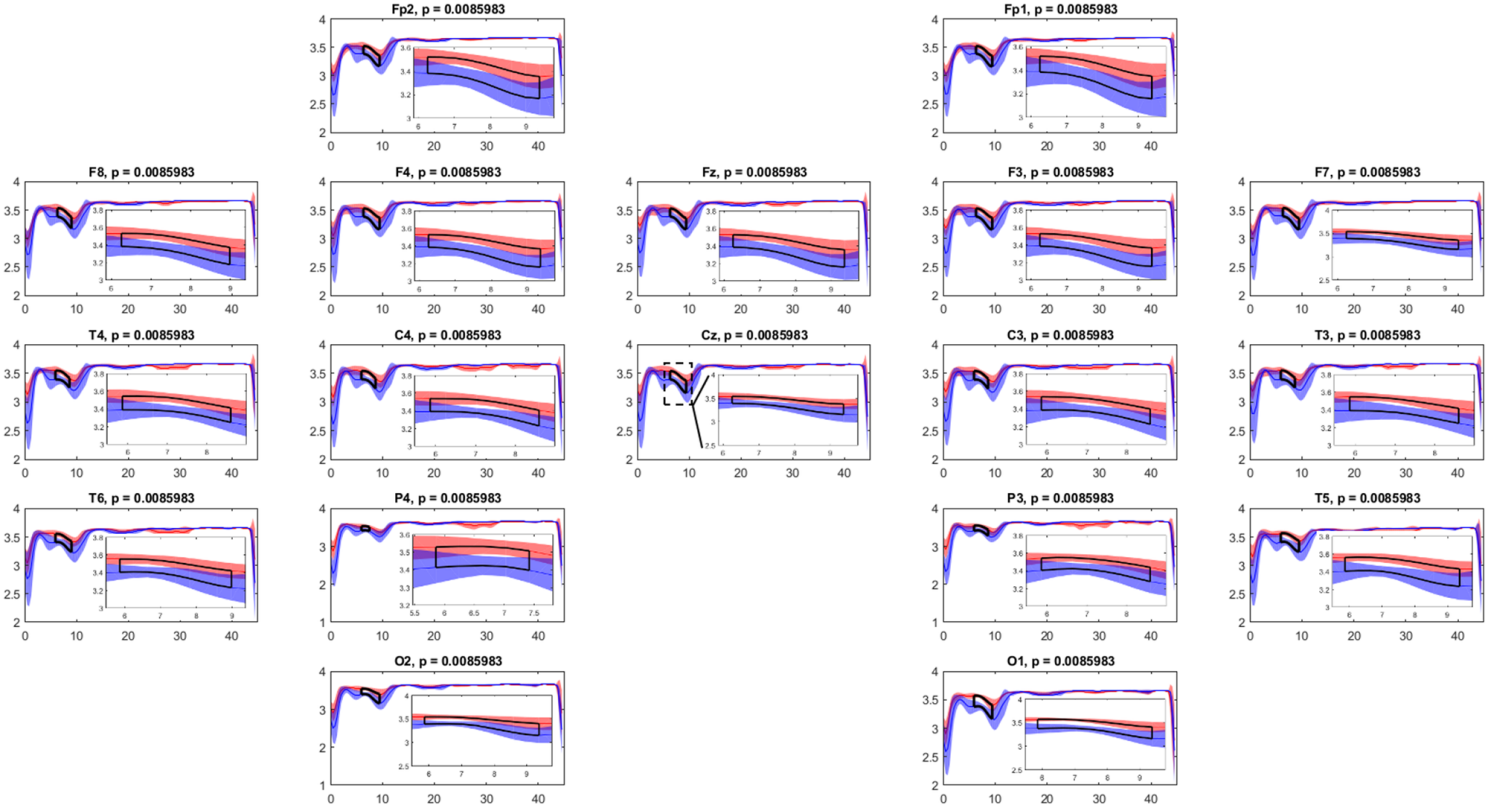
Result of the permutation test. Mean and confidence interval of the LSE for epileptic patients group 1 and group 2 in red and blue respectively. Interval of statistical significance in black. We show zoomed windows of significant frequencies overlapping the entire spectral range at each electrode. There exists significance in all channels at *p* = 0.0086 at least in the range of frequencies [6.25–12.89)Hz.

In Fig 4 we show the result obtained by running the cluster-based permutation test on the LSE samples of the two groups of epileptic patients. The figure depicts the LSE values corresponding to each frequency from 0 to 45Hz. We plot the mean and confidence interval of the LSE for epileptic patients group 1 in red, the mean and confidence interval of the LSE for epileptic patients group 2 in blue and the interval of significance (if it exists) in black. In this test, only 42 out of 5000 partitions had a cluster-level statistic (summed *t*-values) greater than or equal to that of the main cluster. The exact *p*-value is obtained as 43/5001, which accounts for the original cluster configuration (there is at least 1 random configuration with the same statistic as the original). From the figure, it is evident that all electrodes are significant at *p* = 0.0086 at least in the range of frequencies [6.25–7.89 + *w*)Hz = [6.25–12.89)Hz, well below the typically required Monte Carlo alpha-level of 0.025 (for a two-tail test). Conversely, by considering the entire spectrum to estimate the SSE (i.e. following the classical approach) we do not detect any significant difference between groups (Mann-Whitney U-test, *z* = 1.3229, *p* = 0.1859).

### Classification analysis

**Fig 5:**
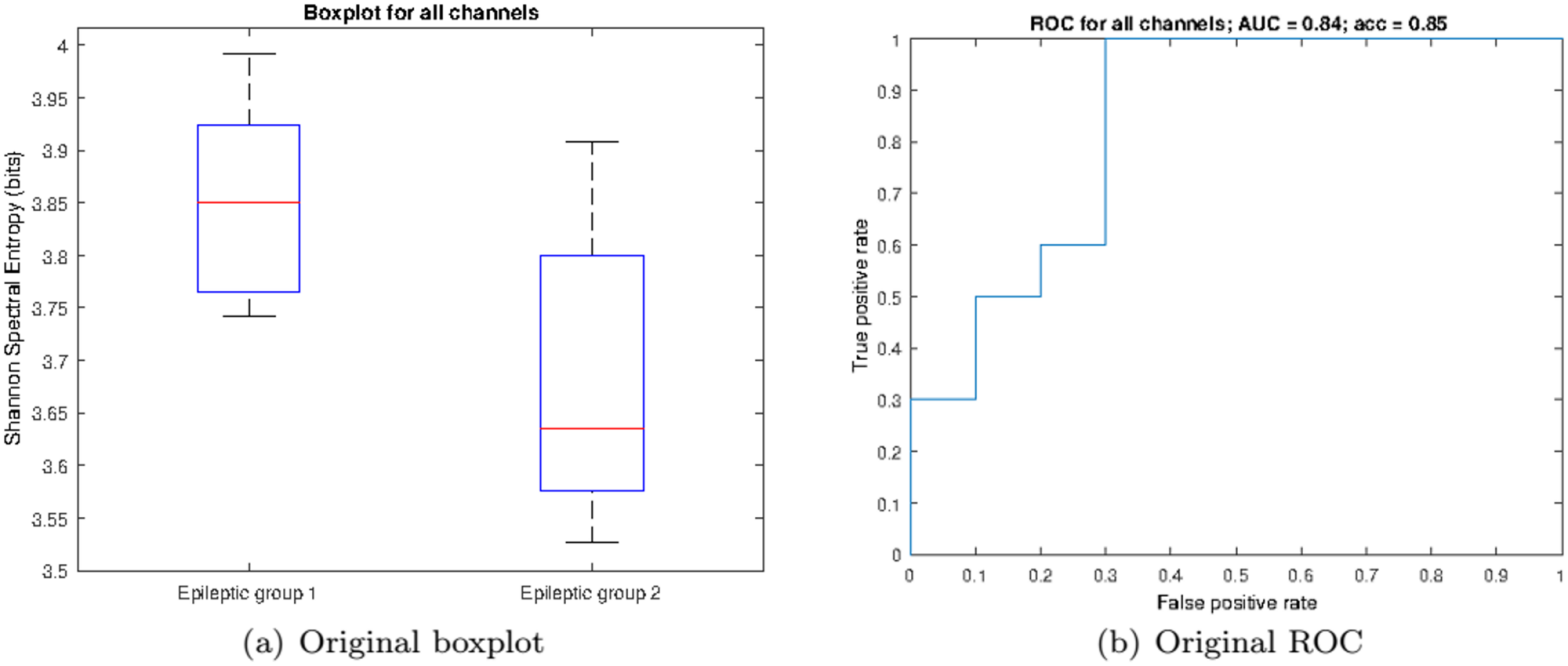
Classification analysis based on all significant electrodes. (a) Boxplot and (b) ROC curve corresponding to the classification analysis of the two groups of epileptic patients. Red lines in the boxes mark the median and edges the 25th and 75th percentiles of the data.

Once the statistical analysis established the channel and frequency limits of significance when comparing the two groups, we analyzed the classification potential of the SSE in the aforementioned limits, i.e. in between 6.25 and 12.89Hz for all channels. Fig 5 shows a boxplot representation of the averaged SSE (across the whole set of channels and frequencies in the detected range) for both groups of epileptic patients. While patients in group 1 are characterized by a median SSE value of 3.85, patients in group 2 present a median of 3.63, thus leading to statistically significant differences (Mann-Whitney U-test, *z* = 2.5324, *p* = 0.01137).

**Fig 6:**
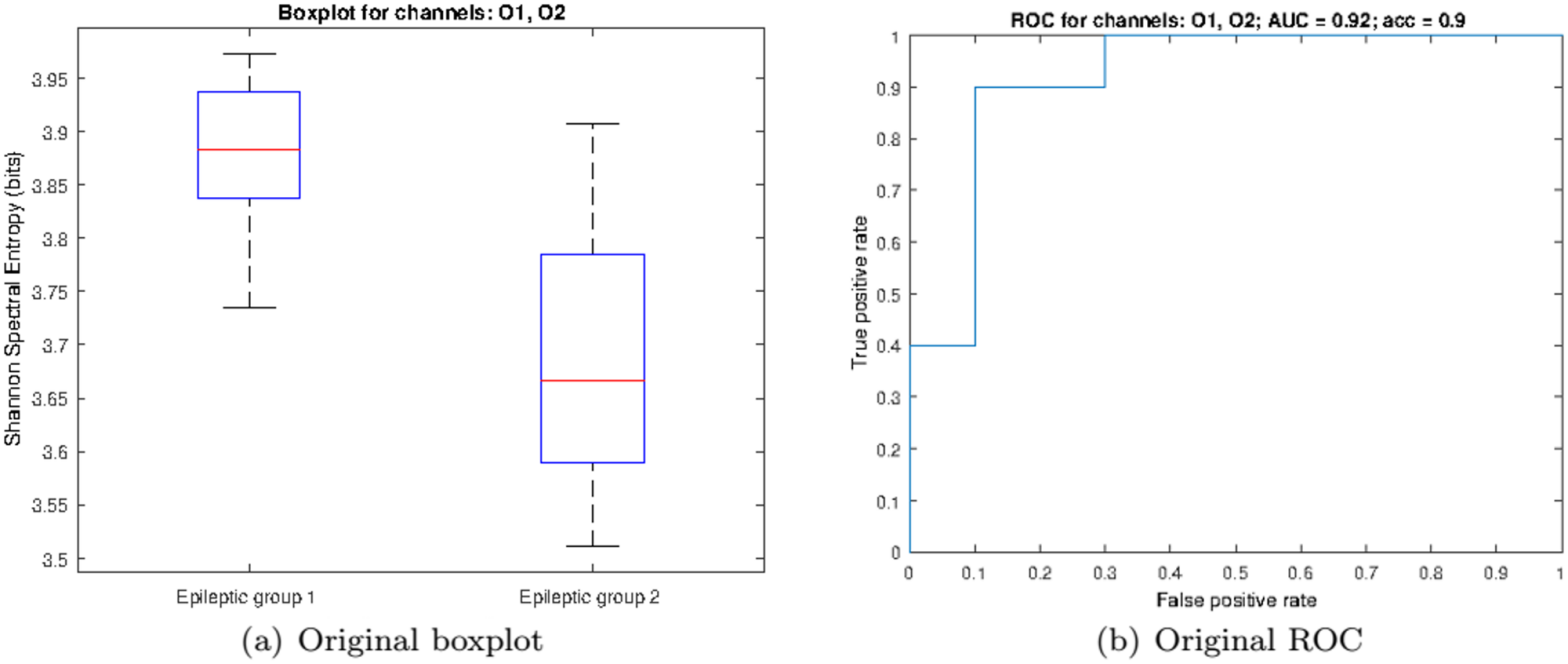
Classification analysis for electrodes O1 and O2. (a) Boxplot and (b) ROC curve corresponding to the classification analysis of the two groups of epileptic patients using values from channels O1 and O2. Red lines in the boxes mark the median and edges the 25th and 75th percentiles of the data.

**Fig 7:**
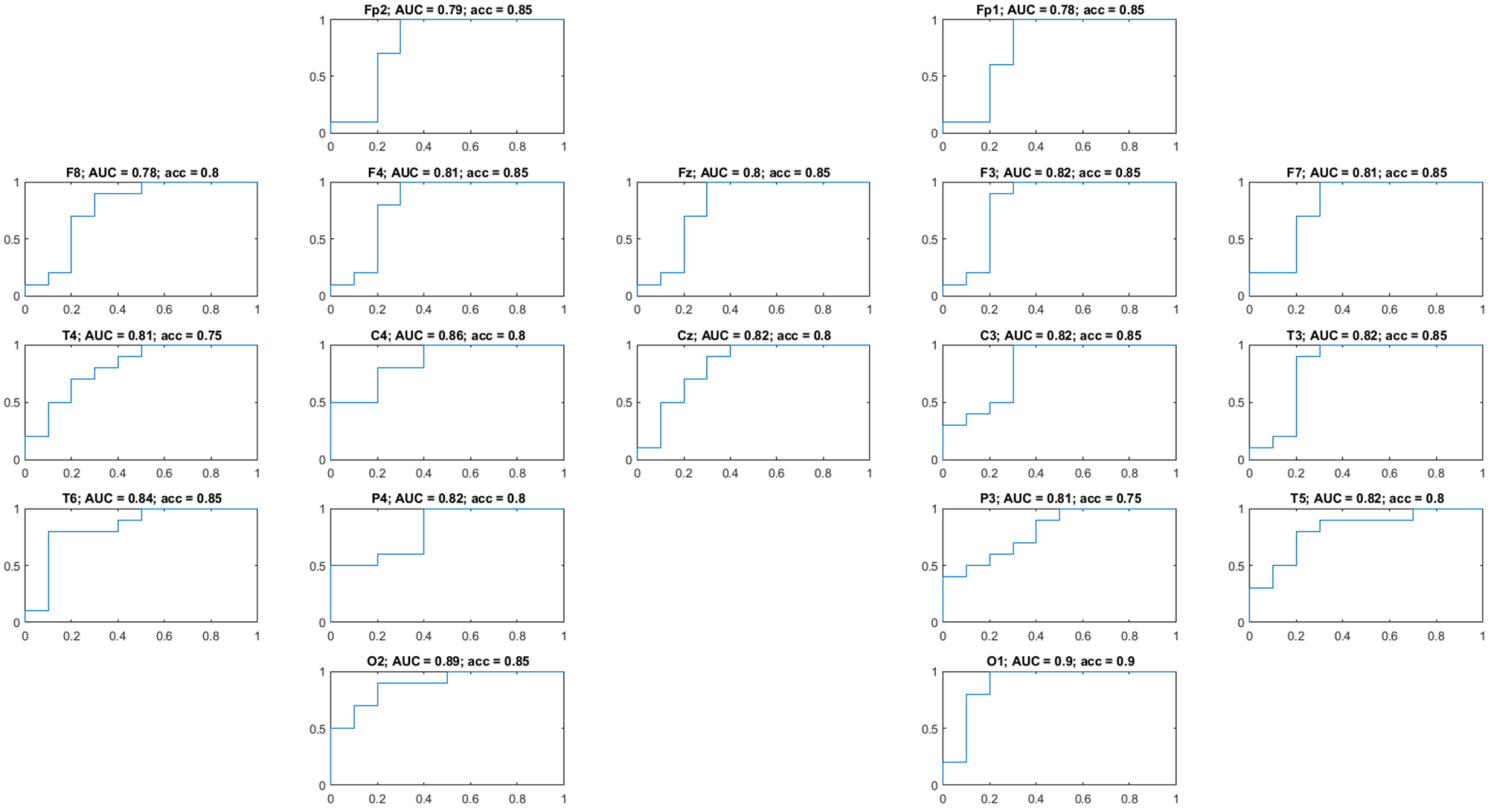
Results of the ROC analysis for all channels. The best channels are O1 and O2 since we want to minimize the false positive rate while having a good true positive rate.

We then evaluated the “diagnostic” ability of the LSE by carrying out a two-class ROC analysis to determine group discrimination. By directly using all channels, we obtained the ROC as depicted in Fig 5(b) characterized by an AUC = 0.84 (area under the curve) and an accuracy of 85% at the optimal working point (0.3, 1.0) for a threshold *T* = 3.7423 (bits). However, it is possible to improve classification by carefully choosing a subset of all available channels. In fact, by averaging the SSE at channels O1 and O2, we obtain better group separation as depicted in the boxplot of Fig 6(a) and further corroborated by the ROC of Fig 6(b), which is characterized by an AUC = 0.92 and an accuracy of 90% at the optimal working point (0.3, 1.0) for a threshold *T* = 3.7876 (bits). In order to determine such best subset, we performed a two-class ROC analysis per channel, as shown in Fig 7, where the optimal individual ROCs are found to be for channels O1 and O2.

### Reproducibility

To further validate our results, we performed a 5-fold cross-validation. We tested the reproducibility of our experiments, both for the entire set of channels and also for the optimal subset of electrodes. In Table 2 we summarize the results obtained for the optimal set of electrodes (O1 and O2), and show that all subsets are classified satisfactorily with average true positive rate equal to 86% and true negative rate equal to 76% (i.e. 81% mean accuracy).

**Table 2:** 5-fold cross-validation (80/20).

Fold	TPR	TNR	*T* (bits)
#1	100%	60%	3.8486
#2	80%	70%	3.8063
#3	80%	80%	3.8042
#4	80%	100%	3.8121
#5	90%	70%	3.8409
Average	86%	76%	3.8224

TPR (true positive rate / sensitivity): percentage of epileptic patients from group 1 correctly identified. TNR (true negative rate / specificity): percentage of epileptic patients from group 2 correctly identified

### Dynamics of primary generalized epilepsy

**Fig 8:**
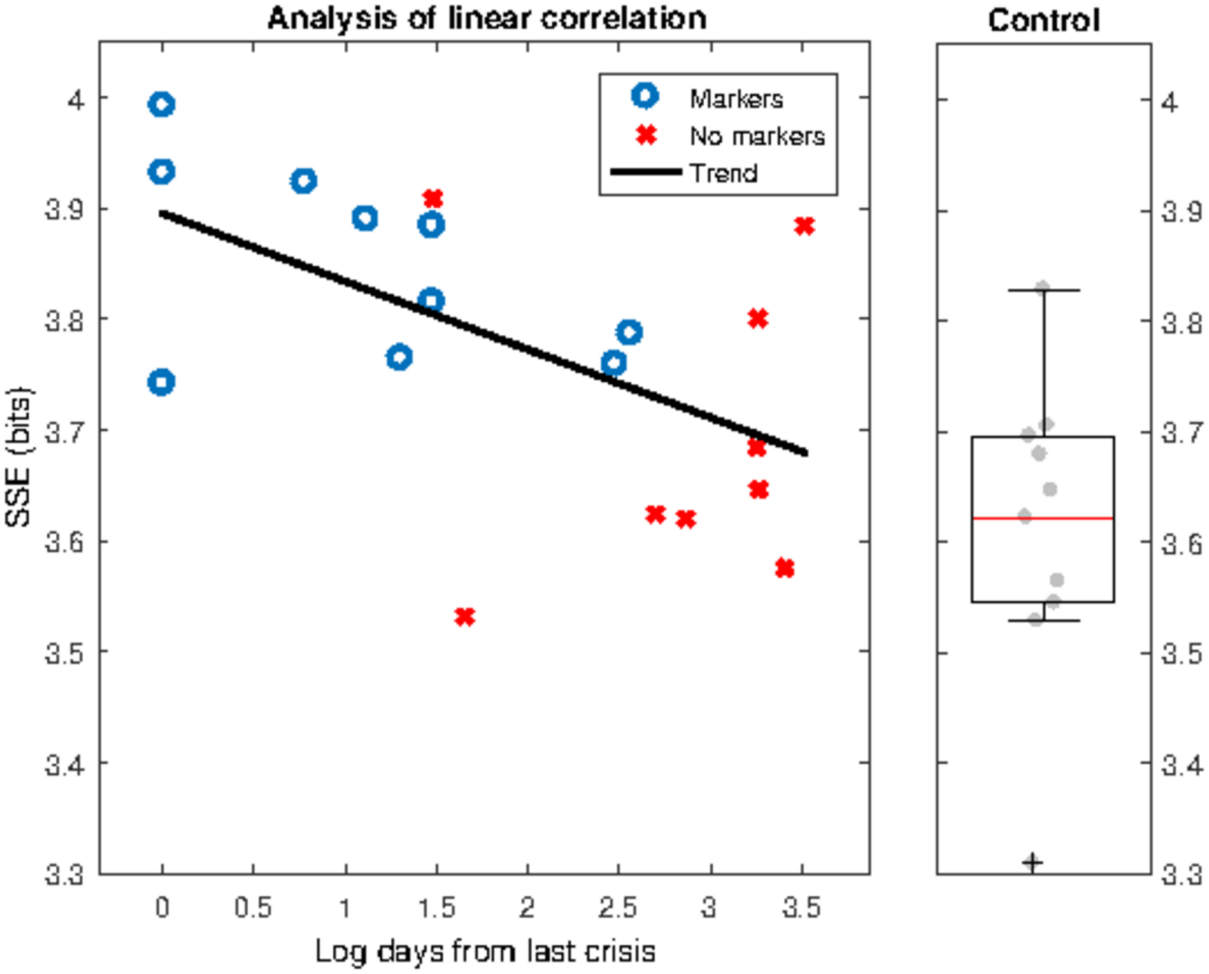
Linear fit of SSE vs time elapsed (*log*_10_) from last crisis (left) and boxplot of the SSE of healthy subjects in the band of frequencies of interest (right). The fit suggests that the SSE decreases as the time from the last crisis increases. Moreover, it should be noted that the SSE values seems to asymptotically converge in time to the levels of control subjects.

Next, and motivated by the evidence that most patients from group 2 (patients without epileptiform activities in the background EEG of any of the recordings available) had had their last crisis longer ago than those from group 1 (patients with epileptiform activities in the background EEG of portions of recordings not used for this study), we inspected the correlation between the SSE in the frequency band of interest and the time elapsed from the last crisis. To do that, we employed a linear regression model to fit the SSE (in bits) to the time from last crisis (in log days scale). Fig 8 suggests the existence of a significant correlation between the SSE and the time elapsed from the last crisis, (*R*^2^ = 0.253, *F*_1,17_ = 5.77, *p* = 0.028; *x*_1_ = −0.057679 bits /log_10_ days, *p* = 0.028; intercept = 3.9016, *p* < 0.001). Indeed, we observe that the SSE values reduce from 3.85 bits to 3.65 bits from time 1 day to ≈ 8 years after seizures, and asymptotically trend to the SSE values of the subjects in the control group (median ∼3.63 bits).

As expected, when comparing the SSE values for the three groups, the statistical analysis shows a significant dependence of the group factor (Kruskal-Wallis one-way ANOVA test, $\chi_{\left(29,2\right)}^{2}=16.1832$, *p* < 0.001). Post hoc analyses revealed that the epileptic group 1 is characterized by significantly higher values of SSE than the epileptic group 2 or the control subjects (multiple comparison test, *p* < 0.05). In contrast, no significant differences between epileptic patients from group 2 and control subjects were found (*p* > 0.05).

All these results suggest that the SSE, measured in the specific frequency range detected by the permutation approach, allows to detect hidden features in the background activities that could be related to the normalization of the EEG activity in those patients with well-controlled epilepsies. Indeed, these values trend to match to those of control subjects recorded in the same conditions.

## Discussion

Several authors have studied detection and prediction of epileptic EEG signals when ictal activity is present in the recordings along with background EEG rhythms (see [4, 5] for specific examples and [6, 7] for reviews on the subject). Traditional basic linear analyses in the time and frequency domains have been complemented or even replaced by more advanced linear and non-linear features, motivated by the inherent non-linearity of the brain. However, epileptic activities are not always present in EEG recordings and, in such situations, the development of techniques based on the analysis of background EEG may be critical. Nowadays, discriminating between healthy and epileptic subjects by visual inspection alone or by applying readily available simple processing methods on EEG rhythms is indeed quite complex, more so in the medical environment. This is likely the reason why, in contrast with the abundant work dedicated to epileptic seizures, publications in the literature that concentrate on studying spontaneous EEG activity of epileptic subjects are scarce [16, 17].

The most relevant features for processing resting state EEG in recent literature are possibly based on information theory. A variety of linear and nonlinear parameters such as spectral entropy [4, 6, 18], wavelet entropy [19], approximate entropy [20], fractal dimension [21] or Lempel-Ziv complexity [5] have been used to study the complexity of magneto- and electroencephalographic signals in patients with epilepsy, as well as in other neurological diseases [22–25].

In this study, we have investigated EEG background activities of patients suffering from primary generalized epilepsy using periods of EEG recordings that presented no epileptiform activity. We have concentrated on an entropy metric because it is an intuitive parameter to measure the complexity of a signal and since it does not depend on absolute scales like the amplitude or the frequency of the signal, which is vital in an EEG application. The SSE metric was proposed by Powell et al. in [26] to calculate the entropy based on the distribution of the power spectrum [27] and applied some 20 years later by Inouye et al. for quantifying irregularity of EEGs [11]. Several authors have applied SSE to the analysis of ictal recordings [4, 18], revealing that the SSE values seem to be lower during ictal periods (seizures) than in normal EEG segments [28, 29].

Here, we have proposed local spectral entropy (LSE), computed as the SSE within a specific range of frequencies, and investigated the convenience of such metric to quantify the complexity of the EEG signal for consecutive spectral bands. The LSE, coupled with a cluster-based permutation analysis, has allowed us to detect the optimal combination of frequencies and channels that better differentiates the two group of epileptic patients. More specifically, the statistical analysis conducted on the LSE-channel pairs revealed significant differences at all available channels when estimated in between 6.25 and 12.89Hz. Indeed, patients with EEG signals containing epileptic markers in other portions of their recordings show significantly larger LSE values than patients with apparently normal background activity. Moreover, by averaging the LSE corresponding to the channels with greatest individual ROC values, (O1 and O2) we were able to classify groups of epileptic patients with 90% accuracy.

When compared to the values estimated from a control group of healthy subjects, the LSE values from both groups of epileptic patients are larger. However, differences only reach the statistical threshold when compared with the LSE values of the patients with epileptic signatures. Patients without apparent abnormalities in the EEG present LSE values in between those of the control subjects and those of the epileptic patients with markers. Furthermore, when considering the elapsed time from the last seizure, results suggest that the stage of primary generalized epilepsy can be related to the complexity of resting state brain dynamics in a direct manner; there is a significant correlation between the LSE in the [6.25–12.89) Hz range and the time elapsed from the last seizure. Specifically, the LSE measured in epileptic patients decreases as the time from the last crisis increases and asymptotically trends to converge to the LSE values measured in the control group of healthy subjects. This is a relevant finding on its own because, even if we can not propose the LSE as a prediction metric for seizures (there are no clues about a potential increase of the LSE right before the seizure), it could well assist clinicians in the follow-up the patients after the seizure.

Other studies have found that SSE values are lower in background EEGs/MEGs recordings of patients compared to control subjects [5, 16, 17]. Our study differs from these in that our control group of healthy subjects present lower SSE values. But, bear in mind that, on the one hand, metrics are not exactly the same in our study compared to other studies and, on the other hand, our analysis is restricted to a narrow band of frequencies that has been selected within an statistical framework devoted to detect the most suitable combination of frequencies and channels for the classification task. In fact, by considering the entire spectrum, we do not detect any significant differences between groups. Future lines of research could consider other complexity metrics that have been successful in the literature, such as approximate entropy, fractal dimension, Lempel-Ziv complexity or the wavelet turbulence of [30] to further validate our results and to assess the predictive value of this framework for the anticipation of seizures.

## Conclusions

We have studied and compared the SSE estimated from background EEG activity of two groups of patients suffering from primary generalized epilepsy and a control group of healthy subjects recorded with the same setup. We have found that, computed in the [6.25–12.89) Hz range, the SSE is able to detect hidden characteristics of the EEG complexity that allows to differentiate between patients with epileptic markers in the EEG from patients with apparently normal EEG. When compared with a group of healthy subjects, epileptic patients (1) showed larger values of LSE that (2) were inversely related to the elapsed time from the last seizure. These findings suggest that, when patients are well controlled after the seizures, the complexity of the resting brain dynamics gradually evolves to a less complex state, with a trend to reach a state that characterizes healthy subjects. Additional studies including a complete follow-up of patients before, during and after an epileptic seizure could serve not only to confirm this hypothesis but, potentially, also to assess the validity of the current approach in the task of understanding and monitoring how the brain transits to and from seizures.
